# The ribosomal transcription units of *Haplorchis pumilio* and *H. taichui* and the use of 28S rDNA sequences for phylogenetic identification of common heterophyids in Vietnam

**DOI:** 10.1186/s13071-017-1968-0

**Published:** 2017-01-09

**Authors:** Thanh Hoa Le, Khue Thi Nguyen, Nga Thi Bich Nguyen, Huong Thi Thanh Doan, Do Trung Dung, David Blair

**Affiliations:** 1 Institute of Biotechnology, Vietnam Academy of Science and Technology, 18. Hoang Quoc Viet Rd,, Cau Giay Hanoi, Vietnam; 2 Department of Parasitology, National Institute of Malariology, Parasitology and Entomology, Luong The Vinh Rd,, Thanh Xuan Hanoi, Vietnam; 3 College of Science and Engineering, James Cook University, Townsville, Australia

**Keywords:** Ribosomal transcription unit, *Haplorchis pumilio*, *Haplorchis taichui*, Heterophyidae, 28S rDNA sequence, Phylogeny

## Abstract

**Background:**

Heterophyidiasis is now a major public health threat in many tropical countries. Species in the trematode family Heterophyidae infecting humans include *Centrocestus formosanus*, *Haplorchis pumilio*, *H. taichui*, *H. yokogawai*, *Procerovum varium* and *Stellantchasmus falcatus.* For molecular phylogenetic and systematic studies on trematodes, we need more prospective markers for taxonomic identification and classification. This study provides near-complete ribosomal transcription units (rTU) from *Haplorchis pumilio* and *H. taichui* and demonstrates the use of 28S rDNA sequences for identification and phylogenetic analysis.

**Results:**

The near-complete ribosomal transcription units (rTU), consisting of 18S, ITS1, 5.8S, ITS2 and 28S rRNA genes and spacers, from *H. pumilio* and *H. taichui* from human hosts in Vietnam, were determined and annotated. Sequence analysis revealed tandem repetitive elements in ITS1 in *H. pumilio* and in ITS2 in *H. taichui.* A phylogenetic tree inferred from 28S rDNA sequences of 40 trematode strains/species, including 14 Vietnamese heterophyid individuals, clearly confirmed the status of each of the Vietnamese species: *Centrocestus formosanus, Haplorchis pumilio*, *H. taichui*, *H. yokogawai*, *Procerovum varium* and *Stellantchasmus falcatus*. However, the family Heterophyidae was clearly not monophyletic, with some genera apparently allied with other families within the superfamily Opisthorchioidea (i.e. Cryptogonimidae and Opisthorchiidae). These families and their constituent genera require substantial re-evaluation using a combination of morphological and molecular data. Our new molecular data will assist in such studies.

**Conclusions:**

The 28S rDNA sequences are conserved among individuals within a species but varied between genera. Based on analysis of 40 28S rDNA sequences representing 19 species in the superfamily Opisthorchioidea and an outgroup taxon (*Alaria alata*, family Diplostomidae), six common human pathogenic heterophyids were identified and clearly resolved. The phylogenetic tree inferred from these sequences again confirmed anomalies in molecular placement of some members of the family Heterophyidae and demonstrates the need for reappraisal of the entire superfamily Opisthorchioidea. The new sequences provided here supplement those already available in public databases and add to the array of molecular tools that can be used for the diagnosis of heterophyid species in human and animal infections.

## Background

Many members of the trematode family Heterophyidae Odhner, 1914, use fishes as intermediate hosts and humans as definitive hosts [1, 2]. Six species in particular, *Centrocestus formosanus*, *Haplorchis pumilio*, *H. taichui*, *H. yokogawai*, *Procerovum varium* and *Stellantchasmus falcatus* [2, 3] are among the most clearly recognized human pathogens and mostly occur in eastern Asia including China, the Philippines, Korea, Taiwan, Thailand, Laos, Cambodia and Vietnam [3–11]. Heterophyidiasis caused by these and related species has now become a major public health threat, not only in Asia but in parts of Africa and the Americas [3, 5, 10, 12, 13]. Humans acquire heterophyid infection by consumption of undercooked or raw freshwater fishes containing infective metacercariae [3, 14]. Infection with multiple species is frequently reported in Vietnam and elsewhere [3, 5, 7, 9, 14].

DNA sequences are commonly used for molecular diagnosis and systematic/phylogenetic studies. Although markers are often chosen from the mitochondrial genome, sequences from the nuclear ribosomal transcription unit (rTU) (including 18S, ITS1, ITS2 and 28S) are particularly useful and reliable for this purpose [10, 15–22]. A single rTU consists of three coding regions (the 18S, 5.8S and 28S rRNA genes) separated by two internal transcribed spacers (ITS1 and ITS2) [17]. Short external transcribed spacers (ETS) are found 5' of the 18S gene and 3' of the 28S gene. Adjacent rTUs in the ribosomal array are separated by a long non-transcribed intergenic spacer (IGS) region [17, 23, 24]. Sequences from various portions of the rTU (18S, ITS1, ITS2 and 28S) have been widely used for inference of phylogenetic relationships and taxonomic clarification within and between many trematode families (e.g. [15, 18, 22, 25–31]). Sequences of complete or near-complete rTUs are only available for a few species of trematode [12, 16, 20, 32, 33]. Clearly, however, such data will be valuable for many kinds of comparative analysis, including systematics/phylogenetics and studies on intra- and interspecific or even intra- and interindividual variation in trematodes [15, 18, 20, 34, 35]. In particular, these data are needed for the large family Heterophyidae, which comprises more than 30 genera, many containing species infecting humans [1, 2, 12, 15, 34]. Heterophyid species in Vietnam have well been described epidemiologically and morphologically, but molecular data useful for diagnosis and identification, as well as taxonomy, are still limited [5–7, 9, 14].

The aim of this paper is to present the sequence of near-complete ribosomal transcription units from *Haplorchis pumilio* and *H. taichui*, commonly found in humans. Portions of the 28S rRNA gene from other heterophyids infecting humans in Vietnam are also presented, i.e. *Centrocestus formosanus*, *Haplorchis yokogawai*, *Procerovum varium* and *Stellantchasmus falcatus*. The data will be used to explore the phylogenetic positions of these genera in the family Heterophyidae and in the class Trematoda.

## Methods

### Heterophyid samples

Metacercariae of *Haplorchis* spp. and *Centrocestus* spp. were collected from fish species (common carp, *Cyprinus carpio*, and grass carp, *Ctenopharyngodon idellus*) and cercariae from freshwater snails (*Melanoides tuberculata*) in Nam Dinh Province [8, 14] (Table 1).

**Table 1:** Summary data for the heterophyids used in the phylogenetic analysis and molecular identification

Sequence code	Life-cycle stage	Host	Province	Reference	GenBank No.	Identification
CfoHG2	Adult	Human	Ha Giang	This study	KY369153	*Centrocestus formosanus*
CspMND2	Metacercaria	Fish	Nam Dinh	[14]	KY369154	*Centrocestus formosanus*
HPU8QT	Adult	Human	Quang Tri	This study	KY369155	*Haplorchis pumilio*
HPU6HG	Adult	Human	Ha Giang	This study	KY369156	*Haplorchis pumilio*
HspCeS1	Cercaria	Snail	Nam Dinh	[8]	KY369157	*Haplorchis pumilio*
HpDzH	Adult	Human	Nam Dinh	This study	KX815125	*Haplorchis pumilio* ^a^
HTA2HG	Adult	Human	Ha Giang	This study	KY369158	*Haplorchis taichui*
HTAQT3	Adult	Human	Quang Tri	This study	KX815126	*Haplorchis taichui* ^a^
HspYOK	Metacercaria	Fish	Nam Dinh	This study	KY369159	*Haplorchis yokogawai*
An394	Cercaria	Snail	Nam Dinh	[8]	KY369160	*Haplorchis yokogawai*
HspND	Adult	Human	Nam Dinh	This study	KY369161	*Procerovum varium*
SfND	Adult	Human	Nam Dinh	This study	KY369162	*Stellantchasmus falcatus*
SfQN1	Adult	Human	Quang Ninh	This study	KY369163	*Stellantchasmus falcatus*
SfQN2	Adult	Human	Quang Ninh	This study	KY369164	*Stellantchasmus falcatus*

^a^ *Haplorchis pumilio* and *H. taichui* samples chosen for sequencing the near complete ribosomal transcription unit

Adults of *Centrocestus* spp., *Haplorchis* spp., *Procerovum* spp. and *Stellantchasmus* spp., originating from Ha Giang, Nam Dinh, Quang Tri and Quang Ninh Provinces, in the north of Vietnam, were collected directly from feces of naturally infected humans after treatment with praziquantel and purgation by magnesium sulfate (MgSO_4_) [5, 14] (Table 1). Each adult worm, unstained or stained with acetic carmine, was morphologically identified to species by light microscopy [3, 5, 14]. Up to ten worms of each species recovered per human were individually fixed in 70% ethanol; one or two worms of each species were subjected to molecular analysis. The samples HTAQT3 of *Haplorchis taichui* and HpDzH of *H. pumilio*, collected from people in Quang Tri and Nam Dinh Provinces, respectively, were chosen for amplification and sequencing of the rTU. Only the 28S region was amplified and sequenced from other species for molecular identification and phylogenetic analysis (Table 1).

### Genomic DNA extraction, primers and amplification

Total genomic DNA was extracted from individual cercariae, metacercariae or adult specimens using the GeneJET™ Genomic DNA Purification Kit (Thermo Fisher Scientific Inc., MA, USA), according to the manufacturer’s instructions. Genomic DNA was eluted in 50 μl of the elution buffer provided in the kit and stored at -20 °C. The DNA concentration was estimated using a GBC UV/visible 911A spectrophotometer (GBC Scientific Equipment Pty. Ltd., Braeside VIC, Australia) and diluted to a working 50 ng/μl: 2 μl were used as template in a PCR of 50 μl volume.

All rTU-universal primers, used both for amplification and sequencing the rTU of *H. pumilio* and *H. taichui*, are listed in Table 2. Primers UD18SF/U3SR amplified the 18S and ITS1 region and U3SF/1500R amplified the ITS2 and 28S region. The primer pairs U18SF/U18SR and U28SF/U28SR, were used for obtaining major fragments of ribosomal 18S or 28S, respectively. These primers were also used as sequencing primers, as were additional internal primers (Table 2).

**Table 2:** Primers for amplification and sequencing of the ribosomal transcription unit

Primer name	Sequence (5'–3')	Length (bp)	Tm (°C)	Target gene	Reference
UD18SF	AACCTGGTTGATCCTGCCAG	20	59	18S (F)	[15]
NS1F	GTAGTCATATGCTTGTCTC	19	48	18S (F)	This study
U18SF	GCGAATGGCTCATTAAATCAGC	22	57	18S (F)	This study
U18S2R^a^	GGTTCTGTTCTAATAAATCCAC	22	50	18S (R)	This study
U18SAR^a^	CCGTCGCCGACACAAGGCCGAC	22	67	18S (R)	This study
NS2F^a^	GCAAGTCTGGTGCCAGCAGCC	21	66	18S (F)	This study
NS2R^a^	GGCCTGCTTTGAGCACTC	18	59	18S (R)	This study
U18S2F	TCGTGACTGGGATCGGGGC	19	64	18S (F)	This study
NS5F^a^	TGAATGGTTTAGCAAGGTCCTCGG	24	61	18S (F)	This study
U18SR^a^	GGAACCAATCCGAGGACCTTGC	22	63	18S (R)	This study
NS8R^a^	CACCTACGGAAACCTTGTTACGACTT	26	60	18S (R)	This study
U3SF	GGTACCGGTGGATCACTCGGCTCGTG	26	67	5.8S (F)	This study
U3SR	CGACCCTCGGACAGGCG	17	64	5.8S (R)	This study
U28SF^a^	CTAACAAGGATTCCCTTAGTAAC	23	52	28S (F)	This study
U28S2R^a^	ACAACCCGACTCCAAGGTC	19	59	28S (R)	This study
U28F^a^	TCGGAGACGGCGGCTTG	17	63	28S (F)	This study
U28S2F^a^	ATCACCGGCCCGTCCCATG	19	65	28S (F)	This study
U28SR	GTCTTTCGCCCCTATACTCAC	21	57	28S (R)	This study
1500R	GCTATCCTGAGGGAAACTTCG	21	57	28S (R)	[15]

*Abbreviations* *F* forward, *R* reverse, *Tm* melting temperature

^a^Primers used for sequencing

PCR reactions of 50 μl were prepared using 25 μl of DreamTaq PCR Master Mix (2×) (Thermo Fisher Scientific Inc., MA, USA) and 2 μl DNA template (50 ng/μl), 2 μl of each primer (10 pmol/μl), 2 μl DMSO (dimethyl sulfoxide) and 17 μl H_2_O. All PCRs were performed in a MJ PTC-100 thermal cycler with initiation at 94 °C for 5 min, followed by 35 cycles consisting of denaturation for 30 s at 94 °C, annealing at 56 °C for 30 s, extension at 72 °C for 6 min; and a final extension at 72 °C for 10 min. The PCR products (10 μl of each) were examined on a 1% agarose gel, stained with ethidium bromide, and visualized under UV light (Wealtec, Sparks, NV, USA).

The amplicons were eluted from the gel and subjected to direct sequencing by primer-walking in both directions.

### Annotation and phylogenetic analysis

Boundaries of ribosomal 18S, 5.8S and 28S genes were determined by alignment, using the Clustal X program [36], with known ribosomal DNA sequences inferred from complete or near-complete rTU sequences available in the GenBank database or previous publications, i.e. for *Euryhelmis costaricensis* (GenBank: AB521797); *Isthmiophora hortensis* (AB189982); *Paragonimus kellicotti* (HQ900670); *Paramphistomum cervi* [33]; and some partial rTUs including *Centrocestus* sp. (AY245699); and *Haplorchis pumilio* (AY245706) and *Haplorchis taichui* (AY245705) [12]. For internal transcribed spacers, ITS1 was recognized as the region located between 18S and 5.8S and ITS2 as between and 5.8S and 28S, respectively. Tandem repeats (TRs) were detected in the ITS1 or ITS2 using the Tandem Repeat Finder v3.01 [37].

Newly obtained partial 28S sequences (approximately, 1,100 nucleotides) of 14 Vietnamese heterophyids and 25 additional sequences, representing species of all three families of the superfamily Opisthorchioidea available in GenBank, and including another 17 sequences from members of the family Heterophyidae, were aligned using GENEDOC2.7 (available at: http://iubio.bio.indiana.edu/soft/molbio/ibmpc/genedoc-readme.html) (Tables 1 and 3). Also included in the alignment was *Alaria alata* (family Diplostomidae) as an outgroup species. The alignment was trimmed to the length of the shortest sequence, saved in FASTA format and imported into the MEGA6.06 software. To examine the phylogenetic position of the Vietnamese heterophyids relative to other trematodes, a phylogenetic tree was reconstructed (see list of sequences in Tables 1 and 3) using maximum likelihood (ML) analysis with the general time reversible (GTR) + G+ I model (gamma rate heterogeneity and a proportion of invariant sites). This model was given the best Bayesian information criterion score by MEGA. Confidence in each node was assessed using 1,000 bootstrap resamplings [38]. A Bayesian analysis was also conducted using MrBayes v3.2 [39] and the same model of sequence evolution. Five million generations were performed (two parallel runs, each with four chains), more than required for the standard deviation of the splits frequencies to fall below 0.01. Plots indicated that convergence was approached after fewer than 1,000,000 generations. The first 1,000,000 cycles were therefore discarded as ‘burn-in’ and trees sampled every 1,000 generations.

**Table 3:** Summary data for the 28S rDNA sequences for heterophyids and other trematodes available on GenBank and used in the phylogenetic analysis and species identification

Family	Country	Species	GenBank no.	Reference
Heterophyidae	Thailand	*Centrocestus formosanus* ^a^	HQ874609	GenBank
	Germany	*Cryptocotyle lingua*	AY222228	[18]
	Japan	*Euryhelmis costaricensis*	AB521797	[32]
	Japan	*Euryhelmis costaricensis*	AB521799	[32]
	Thailand	*Haplorchis pumilio* ^a^	HM004186	[10]
	Thailand	*Haplorchis taichui* ^a^	HM004181	[18]
	Thailand	*Haplorchis yokogawai* ^a^	HM004178	[10]
	Australia	*Haplorchoides* sp.	AY222226	[15]
	Japan	*Metagonimus hakubaensis*	KM061388	[31]
	Japan	*Metagonimus hakubaensis*	KM061389	[31]
	Japan	*Metagonimus katsuradai*	KM061391	[31]
	Japan	*Metagonimus otsurui*	KM061394	[31]
	Japan	*Metagonimus takahashii*	HQ832636	[18]
	Japan	*Metagonimus yokogawai*	HQ832639	[18]
	Thailand	*Procerovum varium* ^a^	HM004182	[18]
	Vietnam	*Stellantchasmus falcatus* ^a^	HM004174	[18]
	Vietnam	*Stellantchasmus falcatus* ^a^	HM004176	[10]
Cryptogonimidae	Sri Lanka	*Acanthostomum* sp.	KC489792	GenBank
	New Caledonia	*Adlardia novaecaledoniae*	FJ788496	GenBank
	USA	*Caecincola parvulus*	AY222231	[15]
	Australia	*Mitotrema anthostomatum*	AY222229	[15]
Opisthorchiidae	Vietnam	*Clonorchis sinensis*	JF823989	[18]
	Vietnam	*Opisthorchis viverrini*	KY369165	This study
	Thailand	*Opisthorchis viverrini*	HM004188	[10]
	Thailand	*Opisthorchis viverrini*	JF823990	[18]
Diplostomidae	Ukraine	*Alaria alata* ^b^	AF184263	[15]

^a^Published sequences for *C. formosanus*, *H. pumilio*, *H. taichui*, *H. yokogawai*, *P. varium* and *S. falcatus* and used in comparisons with those of the Vietnamese heterophyids

^b^Sequence used as the outgroup

## Results

### Structural organization and characteristics of the ribosomal transcription unit of *Haplorchis pumilio* and *H. taichui*

**Fig. 1:**
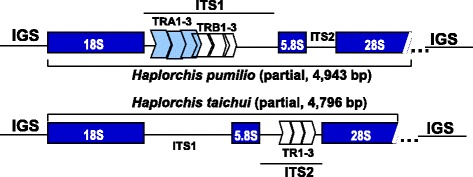
Structural organization of the near-complete ribosomal transcription units for *Haplorchis pumilio* and *H. taichui.* TRA1-3 and TRB1-3 are the tandem repeats in the ITS1 region of *H. pumilio*; TR1-3 are the repeats in *H. taichui*

Near-complete ribosomal transcription units (rTU) from *H. pumilio* and *H. taichui* were determined. The 28S rDNA sequences are conserved among individuals within a species but variable between species and genera. The near-complete rTU is 4,943 nucleotides in length for *H. pumilio*, and 4,796 nucleotides for *H. taichui.* These sequences have been deposited in GenBank under accession nos. KX815125 and KX815126, respectively. We did not sequence the IGS due to the highly repetitive sequences included in this region. The five regions of the rTU are: 18S, ITS1, 5.8S, ITS2 and 28S, structurally organized as usually seen in the ribosomal DNA operon of metazoans (Fig. 1).

In both *H. pumilio* and *H. taichui*, the 18S gene was 1,992 bp in length, and the 5.8S gene was 160 bp long; however, the currently sequenced portion of the 28S gene obtained from *H. pumilio* is 1,397 bp, and that of *H. taichui*, 1,403 bp (Table 4). These lengths represent only a portion of the complete 28S gene (around 3.2–5.5 kb in total for various trematode species [16]). The Vietnamese *H. pumilio* ITS1 region (1,106 bp) contains five complete tandem repeats, (TRA1-2-3, each of 136 bp) and TRB (TRB1-2 each of 123 bp) followed by a partial TRB3 of 84 bp (Table 4; Fig. 1). The ITS1 of the Vietnamese *H. taichui* (797 bp) lacks repeats. In contrast to ITS1, the ITS2 region (444 bp) in *H. taichui* from Vietnam (HTAQT3), possesses three tandem repeats, each of 83–85 bp, while in *H. pumilio* (HpDzH) this region lacks repeats (Table 4; Fig. 1).

**Table 4:** Position of ribosomal genes and internal transcribed spacers in the partially sequenced transcription unit of *Haplorchis pumilio* (4,943 bp) and *H. taichui* (4,796 bp)

Gene/region	Position (5'–3')	Repeat	Size (bp)	Intergenic spacer (bp)	Note
*H. pumilio*					GenBank: KX815125
18S	1–1992		1,992	0	
ITS1	1993–3098		1,106	+66	66 bp to TRA1
	2059–2194	TRA1	136	0	Tandem
	2195–2330	TRA2	136	0	Tandem
	2331–2466	TRA3	136	-91	Overlap with TRB1
	2376–2498	TRB1	123	0	Tandem
	2499–2621	TRB2	123	0	Tandem
	2622–2705	TRB3 (partial)	84	+393	393 bp to 5.8S
5.8S	3099–3258		160	0	
ITS2	3259–3546		288	0	No repeats
28S	3547–4943		1,397		5' partial sequence
*H. taichui*					GenBank: KX815126
18S	1–1992		1,992	0	
ITS1	1993–2779		797	0	No repeats
5.8S	2790–2949		160	0	
ITS2	2950–3393		444	+121	121 bp to TR1
	3071–3155	TR1	85	0	Tandem
	3156–3238	TR2	83	0	Tandem
	3239–3323	TR3	85	+70	Tandem
28S	3394–4796		1,403		5' partial sequence

**Fig. 2:**
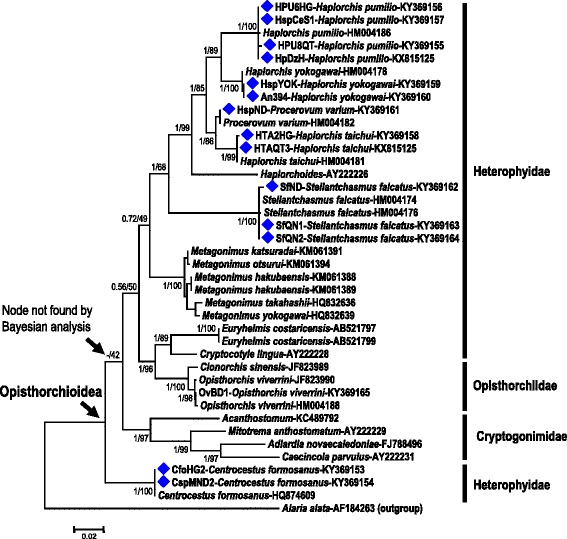
Phylogenetic tree including the six target heterophyid species from Vietnam (*Centrocestus formosanus*, *Haplorchis pumilio*, *H. taichui*, *H. yokogawai*, *Procerovum varium* and *Stellantchasmus falcatus*) and other opisthorchioid trematodes based on partial 28S rDNA sequences (1,100 bp). *Alaria alata* (Diplostomidae) was used as the outgroup taxon. The tree depicted was inferred using maximum likelihood (ML) analysis with the general time reversible (GTR) + G + I model (gamma rate heterogeneity and a proportion of invariant sites) in the MEGA 6.06 package. Support for each node was evaluated using 1,000 bootstrap resamplings [38]. An almost identical tree was found using Bayesian analysis (see text for details). Numbers at nodes are Bayesian posterior support values/ML bootstrap values. The basal node for the superfamily Opisthorchioidea is indicated by an *arrow*. The *scale-bar* indicates the number of substitutions per site. Accession numbers are given at the end of each sequence name

Partial 28S rDNA sequences were obtained from 14 samples of Vietnamese heterophyids representing six species: *Centrocestus formosanus*, *Haplorchis pumilio*, *H. taichui*, *H. yokogawai*, *Procerovum varium* and *Stellantchasmus falcatus* (Table 1). These were aligned with 26 previously published sequences representing 20 species of trematodes in 4 families, including additional representatives of the Heterophyidae (Table 3). The alignment used was 1,100 bp in length. The phylogenetic tree shown in Fig. 2 is based on the maximum likelihood (ML) analysis. Bayesian posterior support values and bootstrap values are shown at relevant nodes. Bayesian and ML trees were almost identical, differing only in the placement of *Centrocestus formosanus*. In the Bayesian tree, this species fell into a clade (posterior support 0.86) with members of the Cryptogonimidae, whereas in the ML tree it was depicted as basal to all other opisthorchioideans (Fig. 2), albeit with low bootstrap support. Sequences of each of our six target heterophyid species were consistently grouped with those of the same species from published sources, thus confirming our morphological identifications. With one exception, species were clustered within their respective genera. The exception was *Procerovum varium*, which was nested among species of *Haplorchis*. Monophyly of the Heterophyidae was not observed. The *Centrocestus formosanus* sequences were grouped either with a sister relationship to the Cryptogonimidae (Bayesian analysis) or basal in the Opisthorchioidea (ML analysis), Sequences of two other heterophyids, *Euryhelmis costaricensis* from Japanese martens (*Martes melampus*) [32] and *Cryptocotyle lingua*, fell into a strongly supported clade (Bayesian posterior support value 1.0 and ML bootstrap support 96%), all other members of which belonged to the family Opisthorchiidae (Fig. 2).

## Discussion

In this study, we have presented sequences of the near-complete ribosomal transcription units (rTUs) for two common species of the family Heterophyidae, *Haplorchis pumilio* and *H. taichui*, which infect humans in Vietnam. The obtained sequences encompass virtually the complete 18S gene (typical length range 1.7–2.9 kb) and almost half of the 28S gene (typical length range 3.3–5.5 kb) [16, 17]. Also obtained were the complete ITS1, 5.8S gene and ITS2 sequences for these species.

We have found repetitive sequences tandemly arranged in the ITS1 of *H. pumilio* and in the ITS2 of *H. taichui*. ITS sequences of both species have been reported from Israel [12]. Israeli *H. pumilio* possessed only two short tandem repeats (30 bp) in their ITS1, in strong contrast to the Vietnamese sequences, in which the ITS1 contained five complete repeats and one incomplete copy. The ITS1 sequences differed substantially in length between Vietnamese and Israeli individuals of the same species, 1,106 *vs* 640 bp in *H. pumilio*; and 797 *vs* 582 bp in *H. taichui*, due to differences in numbers of tandem repeats. These indicate intraspecific polymorphism as reported commonly in trematodes [8, 12, 33]. Likewise, ITS2 showed repetitive sequence differences between individuals from different locations. The presence of repeats in the internal transcribed spacers of trematodes has been reported for several taxa, including those in Schistosomatidae, Opisthorchiidae, Heterophyidae, Paramphistomatidae and others [8, 32, 33, 40]. The presence of repeats, variation in length and sequence variation, within and between species, all contribute to difficulties when trying to align ITS regions. This is particularly so when phylogenetically divergent species are being compared and suggest that this region is not suitable for deep-level phylogenies [17]. At the level of genus and species, however, alignments of ITS sequences have proved valuable for phylogenetic studies and molecular taxonomy [17, 41, 42].

The 18S and 28S rDNA sequences, however, are of considerable value for species identification and phylogenetic analysis [12, 15, 16, 18, 19, 25, 26, 30, 43, 44]. Alignment of these genes is generally straightforward, even among distantly related species, and long repeats do not occur.

The topology of the phylogenetic tree inferred from 40 trematode sequences in this study (Fig. 2) generally agreed well with previous findings. Most genera represented by multiple sequences formed well-supported monophyletic clusters. One striking exception was the sequence of *Procerovum varium*, which rendered *Haplorchis* paraphyletic. This relationship has also been noticed by others (e.g. [10]). Clearly, the definitions of these two genera will need to be revisited. The three families constituting the Opisthorchioidea, the Heterophyidae, Cryptogonimidae and Opisthorchiidae, are very poorly resolved in the tree. The Heterophyidae is not a monophyletic taxon. Indeed, two genera of nominal heterophyids, *Euryhelmis* and *Cryptocotyle*, appear to have closer affinities with the Opisthorchiidae than with the Heterophyidae. This relationship was also found by Thaenkham et al. [34] using 18S rDNA sequences, and by Thaenkham et al. [18] using concatenated 18S and 28S sequences. Paraphyly of the Heterophyidae with respect to the Opisthorchiidae was also demonstrated by [15] using 18S and 28S sequences. An additional heterophyid genus, *Centrocestus*, had an affinity with members of the Cryptogonimidae, or appeared as basal within the Opisthorchioidea (Fig. 2). Such a placement was not supported by analysis of concatenated 18S and 28S sequences by [18]. It is clear that the entire superfamily Opisthorchioidea presents broad systematic and taxonomic challenges to be met in the future using combined morphological and molecular approaches.

## Conclusions

In conclusion, the present study determined and annotated the near-complete ribosomal transcription unit (rTU), consisting of 18S, ITS1, 5,8S, ITS2 and 28S rRNA genes and spacers, from *H. pumilio* and *H. taichui* from human hosts in Vietnam. The ITS1 in *H. pumilio* and ITS2 in *H. taichui* contained tandem repeats. The 28S rDNA sequences are conserved among individuals within a species but variable between species and genera. Based on 28S rDNA sequence analysis of 40 sequences representing 19 species in the superfamily Opisthorchioidea, six common human pathogenic heterophyids, *Centrocestus formosanus*, *Haplorchis pumilio*, *H. taichui*, *H. yokogawai*, *Procerovum varium* and *Stellantchasmus falcatus* were clearly resolved. In addition, the phylogenetic tree inferred from these sequences again confirmed anomalies in molecular placement of some members of the family Heterophyidae and demonstrates the need for reappraisal of the entire superfamily Opisthorchioidea. The new sequences provided here supplement those already available in public databases and add to the array of molecular tools that can be used for the diagnosis of heterophyid species in human and animal infections.

## Abbreviations

ITS: Internal transcribed spacer
rTU: ribosomal transcription unit
TR: Tandem repeat
